# Identification and quantification of fibrotic areas in the human retina using polarization-sensitive OCT

**DOI:** 10.1364/BOE.426650

**Published:** 2021-06-23

**Authors:** Alice R. Motschi, Philipp K. Roberts, Sylvia Desissaire, Markus Schranz, Florian Schwarzhans, Hrvoje Bogunović, Michael Pircher, Christoph K. Hitzenberger

**Affiliations:** 1 Center for Medical Physics and Biomedical Engineering, Medical University of Vienna, Vienna, Austria; 2 Department of Ophthalmology and Optometry, Medical University of Vienna, Vienna, Austria; 3 Center for Medical Statistics, Informatics and Intelligent Systems, Medical University of Vienna, Vienna, Austria; 4 Christian Doppler Laboratory for Ophthalmic Image Analysis, Medical University of Vienna, Vienna, Austria

## Abstract

Subretinal fibrosis is one of the most prevalent causes of blindness in the elderly population, but a true gold standard to objectively diagnose fibrosis is still lacking. Since fibrotic tissue is birefringent, it can be detected by polarization-sensitive optical coherence tomography (PS-OCT). We present a new algorithm to automatically detect, segment, and quantify fibrotic lesions within 3D data sets recorded by PS-OCT. The algorithm first compensates for the birefringence of anterior ocular tissues and then uses the uniformity of the birefringent optic axis as an indicator to identify fibrotic tissue, which is then segmented and quantified. The algorithm was applied to 3D volumes recorded in 57 eyes of 57 patients with neovascular age-related macular degeneration using a spectral domain PS-OCT system. The results of fibrosis detection were compared to the clinical diagnosis based on color fundus photography (CFP), and the precision of fibrotic area measurement was assessed by three repeated measurements in a sub-set of 15 eyes. The average standard deviation of the fibrotic area obtained in eyes with a lesion area > 0.7 mm^2^ was 15%. Fibrosis detection by CFP and PS-OCT agreed in 48 cases, discrepancies were only observed in cases of lesion area < 0.7 mm^2^. These remaining discrepancies are discussed, and a new method to treat ambiguous cases is presented.

## 1. Introduction

Neovascular age-related macular degeneration (nAMD) is a retinal disease posing the elderly population at risk of central vision loss [1,2]. To prevent the progression of the disease, patients are treated with vascular endothelial growth factor suppressor (anti-VEGF) medications [3–5]. However, even with optimal treatment, complications such as foveal atrophy, hemorrhage, or fibrosis may cause irreversible damages to the retina in advanced nAMD. In particular, fibrosis occurs in about half of all cases within two years [6,7].

In order to assess the effect of potential new medications, a robust and objective quantification of fibrosis is needed. Such a monitoring would also be helpful to prognosticate a patient’s future visual acuity and determine the responsiveness of the patient to the treatment, i.e. whether they developed fibrosis or not. Currently, there is no true gold standard imaging technique to quantify the progression of fibrosis in a robust and objective way: clinical evaluations using color fundus photography (CFP), conventional optical coherence tomography (OCT), and fluorescein angiography (FA) are fairly subjective and not reliable.

In CFP, fibrosis appears as obvious white or yellow solid mounds [6]. In OCT images, the accumulations of subretinal hyperreflective material (SHRM) indicate the presence of fibrosis [6–11]. However, SHRM can also be associated with other types of tissues such as blood, exudate, neovascular tissue, lipid or fibrin, and it is not possible to separate it from fibrosis using only conventional OCT [6,8,12–15]. Moreover SHRM is often difficult to distinguish from the retinal pigment epithelium (RPE), which has a similar reflectivity on OCT intensity B-scans, especially if the RPE is disrupted [10,16,17].

To distinguish the RPE from fibrosis and to identify fibrosis within SHRM, additional contrast is needed. Polarization-sensitive optical coherence tomography (PS-OCT) [18,19] is a functional extension of standard OCT, which also analyses the polarization state of light, which is influenced by birefringent or depolarizing material. Early PS-OCT systems were only capable of measuring the phase retardation besides the reflectivity [20,21], but the functionality was soon extended to birefringent axis orientation [22] and more information could be retrieved from Stokes vectors [23,24], Müller matrices [25], and Jones matrices [26,27]. Information on depolarization can be obtained by the polarization scrambling effect of depolarizing tissues, which can be quantified by calculating the degree of polarization uniformity (DOPU) [28].

With respect to polarization, there are three types of tissues in the eye: structures that don’t change the polarization state of light (e.g. the photoreceptor layer) [29], birefringent structures (fibrous structures that exhibit form birefringence, e.g. the sclera, the retinal nerve fiber layer [RNFL], Henle’s fiber layer [HFL], the cornea, and fibrotic tissue) [11,30–32], and depolarizing structures which randomize the polarization of backscattered light (e.g. the RPE) [11,28,29,33]. Birefringent structures decompose an entering light beam into two orthogonally polarized beams which travel at different speeds. Thus, one beam is retarded with respect to the other. This retardation depends on the birefringence and on the thickness of the birefringent structure. The axis orientation in form-birefringent structures is parallel to the angular orientation of the fibers of which the tissue consists. Non-birefringent tissues have no preferential axis orientation, giving rise to a random distribution of axis orientation in PS-OCT axis orientation images.

The birefringence of fibrotic tissue can be exploited to identify fibrosis in retinal tissue. First reported in 2008 [11,33], retinal fibrosis birefringence was later used to identify and quantify fibrotic tissue [34,35]. In these works, the median retardation of depth-integrated A-scans was used to map fibrotic tissue by using a user-set threshold value. While the method worked well in cases with large fibrotic lesions, cases with smaller lesions or thin lesions with low cumulative retardation were frequently missed. The reason is that overlying birefringent tissue like RNFL and HFL distorted the results. If the cut-off retardation threshold was set too low, RNFL and HFL were also segmented, if the threshold was set too high, fibrotic lesions with low retardation were missed.

Another observation can be exploited to achieve a more stable segmentation: the optic axis orientation is a very sensitive measure for the presence of birefringent tissue. Fibrotic tissue shows a well-defined axis orientation, identifiable by tissue patches where the axis locally points into the same direction, while surrounding non-birefringent tissue (polarization maintaining or depolarizing) has no well-defined axis orientation, leading to a random pattern of axis orientation that changes from pixel to pixel. This effect was recently used to identify birefringent tissue in the retina [36] and to detect subretinal fibrosis [37]. In these works, a new parameter, optic axis uniformity (OAxU) was introduced. By visually inspecting OAxU B-scans, in combination with scans of cumulative and of local phase retardation, fibrotic tissue was identified.

In this work, we introduce a new algorithm that expands on our previous work and includes axis uniformity data to detect, segment, and quantify subretinal fibrosis in patients with nAMD. Distorting influences by anterior birefringent structures (cornea, RNFL, HFL) are eliminated by appropriate compensation routines. The algorithm is implemented to work on 3D data sets recorded by a spectral domain PS-OCT system with an integrated retinal tracker that uses a single (circular) polarization state to sample the retina. We performed measurements in 57 eyes of patients with nAMD. In a subset of 15 eyes, we performed repeated measurements to assess the precision (repeatability) of areal segmentation results. Finally, we compare the results of fibrosis detection by PS-OCT with those obtained by color fundus photography.

## 2. Methods

### 2.1 Study protocol

#### 2.1.1 PS-OCT imaging

The instrument used for imaging consists of a custom built spectral domain PS-OCT system based on a Michelson interferometer employing polarization-maintaining (PM) fibers with an integrated retinal tracker that uses a line-scanning laser ophthalmoscope (LSLO) [38]. A sampling beam with a single (circular) polarization state is used. The OCT beam is generated by a superluminescent diode with a center wavelength of 860 nm and a bandwidth of 60 nm, providing an axial resolution of 4.2 µm in tissue and a lateral resolution of ∼20 µm. With an OCT beam of 0.5 mW and an A-scan rate of 70 kHz, a sensitivity of 98 dB is achieved.

The OCT beam is kept at a lower power because simultaneously to PS-OCT imaging, the retina is scanned with a line-shaped LSLO beam with a wavelength of 786 nm at 60 Hz [38]. The LSLO images are used to generate the signals for the retinal tracker [38,39], which requires an LSLO beam power of 0.7 mW. The beam power of PS-OCT is chosen so that the combined beam power of PS-OCT and LSLO fulfill laser safety regulations [40].

The PS-OCT volume scans, consisting of 250 B-scans with 1024 A-scans each (covering a region of size 28° (x) by 21° (y)), are recorded in 4.5 s. The region is approximately centered at the macula and includes parts of the optic nerve head (ONH). Assuming a standard eye length, the 1024 × 250-pixel en-face maps correspond to an area of 8 × 6 mm^2^ on the retina.

#### 2.1.2 Other imaging

To clinically assess the presence of fibrosis, CFP imaging was in addition performed on all patients using the integrated color fundus camera of the MP-3 microperimeter device (Nidek Tokyo, Japan).

### 2.2 Subjects

This study was approved by the local ethics committee and adhered to the tenets of the Declaration of Helsinki. Every patient gave written informed consent prior to study inclusion. 64 patients with nAMD were enrolled in this study. One eye each was selected for imaging.

A good fixation ability and clear ocular media were required to ensure proper image quality. Patients with choroidal neovascularization not secondary to AMD were excluded. A best corrected distance visual acuity between 20/25 and 20/320 was mandatory for inclusion. Three patients had to be excluded due to layer segmentation errors and four patients due to a low signal quality, leading to the number of 57 patients aged 76.9 ± 6.1 years included in this analysis. All 57 eyes have been treated with anti-VEGF injections for one year or longer. Based on CFP judgement by an expert ophthalmologist and reading center-certified grader, 28 out of the 57 eyes ( = 49%) have developed fibrosis.

### 2.3 Data analysis

The data processing consists of several steps which were applied to the PS-OCT data recorded in the 57 eyes, using the same parameters for each measurement.

#### 2.3.1 Standard spectral domain OCT processing of volume data

For each B-scan, the signals recorded at the two channels of the PS-OCT instrument were processed using regular spectral domain OCT data processing, including subtraction of an averaged spectrum, rescaling of spectra from wavelength to wavenumber space, numerical dispersion compensation, and Fourier transform. In a next step, PM fiber length mismatch is compensated, as reported previously [41].

#### 2.3.2 Extraction of polarization data

For each individual B-scan, axis orientation and retardation are extracted as previously described [41] (because of an unknown axis offset introduced by the PM fibers, axis orientation values are relative, i.e., axis orientation changes within an image frame are correct, but absolute axis values would require an additional calibration measurement). DOPU images were computed using an algorithm reported previously [28].

#### 2.3.3 Compensation of birefringence of anterior structures: theoretical aspects

The fibrosis segmentation method used in this work relies on the fact that birefringent tissues exhibit a well-defined optic axis orientation, whereas depolarizing tissues show a random axis orientation. Tissues that maintain the polarization state of backscattered light also show a random axis orientation, if the birefringence of layers anterior to them are properly compensated (otherwise they show the axis orientation “inherited” from the birefringent layers located anterior). The goal of the following data processing steps is to prepare the PS-OCT volume data so that they allow to differentiate between areas of well-defined axis orientation and areas of random axis orientation in the posterior layers of the retina, where fibrotic tissue is located in case of nAMD. There are three birefringent tissue layers that are located anterior to the fibrosis, and whose birefringence has to be compensated before axis uniformity can be analyzed: the cornea, which is always passed by the sampling beam, and the HFL and RNFL for imaging the macular and the more peripheral retina, respectively (the influence of the RNFL is especially pronounced near the ONH).

For compensation of the birefringence of the anterior layers, we use a numerical algorithm that was previously reported in detail for compensation of corneal birefringence [42]. This algorithm is based on the assumption that the layers to be compensated are purely birefringent, i.e. that their diattenuation is negligible, an assumption corroborated by earlier studies [43–45]. The corneal birefringence is generated by a stack of approximately 200 lamellae of collagen fibrils with a thickness of ∼1.5–2.5 µm each, of which the corneal stroma consists. Each lamella can be regarded as a birefringent plate with its slow axis parallel to the fibrils, and successive lamellae having their fibrils usually oriented at large (approximately orthogonal) angles to each other [46]. The birefringence effects of these lamellae largely cancel each other, and a small net birefringence effect, caused by a slight prevalence of one fibril orientation, remains. For a more comprehensive overview of the literature on corneal birefringence, see Ref. [19]. Any stack of linear retarders can be described by a single elliptical retarder [47,48], but it has been shown that the corneal birefringence can be well described by a linear retarder in bovine and porcine cornea [49] and in the central part of most human corneas [50].

The birefringence of HFL and RNFL is caused by the form birefringence generated by the nerve fiber axons of which they consist. In case of the HFL, the axons emerge radially from the foveal cone photoreceptors, giving rise to a doughnut-shaped retardation pattern [30], in case of the RNFL, they emerge approximately radially from the ONH and lead to the ganglion cells in their receptive fields in a well-defined pattern [51,52]. In both cases, the fibers are assumed to have rather constant azimuthal orientation with depth, giving rise to linear birefringence whose orientation varies with transversal position in the ocular fundus.

While all the layers that have to be compensated can thus be approximated as linearly birefringent, their combined effect for a light beam transiting them in single pass is that of an elliptic retarder, as mentioned above. However, in case of imaging in a double pass (round trip) configuration (as with OCT), as a consequence of the Jones reversibility theorem, any combination of birefringent layers with negligible diattenuation can be described by a single linearly birefringent element [48].

For our purpose of analyzing the birefringence of fibrotic tissue that is located behind a stack of other birefringent tissues (cornea, RNFL, HFL), we can therefore describe these layers by an effective single linear retarder that generates, in double pass, the same effective retardation δ_ant,eff_ and has the same effective axis orientation θ_ant,eff_ as the combined anterior layers have in double pass. This effective retarder can then be compensated by a compensating retarder that has the same axis orientation θ_ant,eff_, but a negative retardation of the same size (−δ_ant,eff_) as the effective anterior retarder.

We have previously reported on an algorithm that performs this compensation for a single layer, the cornea, and thereby enables measuring the retardation of the RNFL, the anterior-most birefringent layer of the retina. This algorithm is based on Jones calculus [53], and can be found in Ref. [42]. The same method is now applied for an approximate compensation of the entire effective anterior retarder, including cornea, RNFL, and HFL. (Please note that for a mathematically exact compensation, iterative compensation steps would be required for each layer, however, as discussed in section 4, the difference between approximate and exact compensation is negligible).

According to Eq. (7) of Ref. [42], the Jones vector E_s_ of the sample beam at the detection unit of our PS-OCT system, in case of analyzing the RNFL as the top-most birefringent layer of the retina, can be written as:

(1) $$E_{S}=\frac{1}{2}\cdot J_{Q}\cdot J_{C}\cdot J_{RNFL}\cdot \sqrt{R}\cdot J_{RNFL}\cdot J_{C}\cdot J_{Q}\cdot \left(\begin{array}{c} 0\\ 1 \end{array}\right)$$

where J_Q;C;RNFL_ are the Jones matrices of the linear retarders representing: (Q) the quarter wave plate that converts the vertically polarized input beam (0,1)^T^ into the circular polarized beam illuminating the eye; (C) the cornea; and (RNFL) the retinal nerve fiber layer; and R is the reflectivity of the measured tissue (the RNFL in this case). The Jones matrix of a linear retarder, as a function of its retardation δ and axis orientation θ, is given by:

(2) $$J(\delta ,\theta )=\left\lfloor \begin{array}{cc} \cos^{2}(\theta )+\sin^{2}(\theta )\cdot \exp(-i\delta ) & \cos(\theta )\cdot \sin(\theta )\cdot (1-\exp(-i\delta ))\\ \cos(\theta )\cdot \sin(\theta )\cdot (1-\exp(-i\delta )) & \cos^{2}(\theta )\cdot \exp(-i\delta )+\sin^{2}(\theta ) \end{array}\right\rfloor .$$

If the sampling beam directly hit the RNFL (without passing the cornea), R, δ, and θ of the RNFL could be directly calculated from the measured amplitudes A_H;V_ of the two detection channels (H, horizontal; V, vertical) and from their phase difference ΔΦ by the well-known equations [18,22,42]:

(3) $$R(z)∼A_{H}{\left(z\right)}^{2}+A_{V}{\left(z\right)}^{2}$$

(4) $$\delta (z)=\tan^{-1}\left(\frac{A_{V}(z)}{A_{H}(z)}\right)$$

(5) θ=(π−ΔΦ)/2

where z is the depth coordinate.

Since the measurement is performed through the birefringent cornea, Eqs. (3)–(5) cannot be used directly. Instead, these equations provide the parameters for a joint effective linear retarder J_joint_ that yields, in double pass, the same polarization change as J_C_·J_RNFL_ in double pass. The 3D distribution of δ and θ of J_joint_ is therefore calculated as intermediary step. In a next step, the effect of the cornea has to be compensated. This is done by numerically calculating a compensated Jones vector E_S,comp_ that replaces the directly measured Jones vector E_S_ obtained by Eq. (1).

(6) $$E_{S,comp}=\frac{1}{2}\cdot J_{Q}\cdot J_{comp}\cdot J_{C}\cdot J_{RNFL}\cdot \sqrt{R}\cdot J_{RNFL}\cdot J_{C}\cdot J_{comp}\cdot J_{Q}\cdot \left(\begin{array}{c} 0\\ 1 \end{array}\right)$$

where J_comp_ is the Jones matrix that compensates the cornea, i.e., it has the same axis orientation as the cornea, but its negative retardation: θ_comp_ = θ_C_, δ_comp_ = −δ_C_, where the indices C and comp indicate the cornea and the compensator, respectively. To obtain θ_C_ and δ_C_, the top surface of the RNFL is segmented, and δ and θ are extracted from the 3D data set at this surface, where they are equal to δ_C_ and θ_C_ (since at this depth the cornea is the only birefringent layer that has been passed by the sampling beam). From J_comp_ and J_joint_, we can now obtain the compensated Jones vector E_S,comp_ by Eq. (6), extract A_H;V_ and ΔΦ, and, by applying Eqs. (4) and (5), obtain the compensated 3D distribution of retardation and axis orientation of the RNFL.

To analyze the birefringent properties of the fibrotic tissue located at the posterior side of the retina, we use the same principle. Equation (1) is now expanded to include the effects of the birefringent layers RNFL, HFL, and the fibrotic tissue, described by Jones matrices J_RNFL_, J_HFL_, and J_Fib_, respectively:

(7) $$E_{S}=\frac{1}{2}\cdot J_{Q}\cdot J_{C}\cdot J_{RNFL}\cdot J_{HFL}\cdot J_{Fib}\cdot \sqrt{R}\cdot J_{Fib}\cdot J_{HFL}\cdot J_{RNFL}\cdot J_{C}\cdot J_{Q}\cdot \left(\begin{array}{c} 0\\ 1 \end{array}\right).$$

From the measured E_S_, we now obtain a joint effective linear retarder that is, in double pass, equivalent to J_C_·J_RNFL_·J_HFL_·J_Fib_. Our compensation now has to cover the combined effect of J_C_·J_RNFL_·J_HFL_. For that, we segment a strongly reflecting layer located behind the HFL. This can either be the boundary between inner and outer photoreceptor segments (IS/OS) or, in case of lesions where the IS/OS boundary is destroyed, the top of the brightly reflecting lesion tissue (or SHRM). At this reference surface, we extract the reference values δ_R_ and θ_R_ and obtain our compensation Jones matrix by setting θ_comp_ = θ_R_ and δ_comp_ = −δ_R_, which can now be used to calculate the compensated Jones vector and, finally, δ and θ for the fibrotic tissue, as described above for the case of the cornea compensation.

#### 2.3.4 Boundary segmentation

As mentioned above, for applying the compensation algorithm, we need to find a proper reference surface from which δ and θ can be obtained to calculate the Jones matrix of the compensating element. For purposes of better illustration (and comparison to our previous work), we segment both, the inner limiting membrane (ILM, i.e. the anterior border of the retina)) to demonstrate the situation where only the cornea is compensated, and the IS/OS line (or lesion surface) to demonstrate the full compensation needed for segmentation of fibrotic tissue. The segmentation lines were retrieved from the graph-based intraretinal layer segmentation method The Iowa Reference Algorithms (Retinal Image Analysis Lab, Iowa Institute for Biomedical Imaging, Iowa City, IA) [54,55]

This automatic layer segmentation was compared in 248 B-scans of 9 retinal volume scans with manually performed segmentations to analyze its performance. On average, the automatic segmentation differed by 3.43 pixels (4.77 µm) and 2.37 pixels (3.29 µm) from manual segmentation lines for the ILM and the IS/OS (or lesion surface), respectively.

#### 2.3.5a Compensation of cornea only

**Fig. 1.**
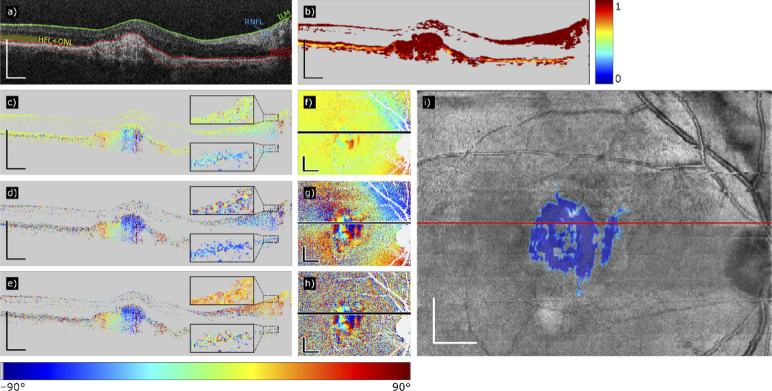
PS-OCT data processing steps to segment subretinal fibrosis demonstrated in the right eye of a patient with fibrosis secondary to nAMD (Patient 39). (a) Intensity B-scan with layer segmentations used as a reference for the compensation of anterior tissue birefringence (green and red solid lines). (b) DOPU image. (c) Uncompensated axis orientation. (d) Cornea-compensated axis orientation (green line in (a) taken as a reference). (e) Fully compensated axis orientation (red line in (a) taken as reference). (f) Average axis orientation en-face maps calculated from the pixels between the solid and the dotted red line in (a), using the uncompensated data. (g) Average axis orientation en-face maps calculated from the pixels between the red solid and the dotted lines, using the cornea-compensated data. (h) Average axis orientation en-face maps calculated from the pixels between the red solid and the dotted lines, using the fully compensated data. (i) Resulting fibrosis segmentation map: pseudo-SLO image superposed with detected areas of fibrosis (blue). Color scheme of the axis orientation: –90° to +90°. Color scheme of DOPU: 0 to 1. Scale bars of the B-scans: 500 µm. Scale bars of the en-face maps: 1 mm.

To demonstrate the effect of compensating only the corneal birefringence, we used the ILM as a reference (green segmentation line in Fig. 1(a)). Figure 1(c) shows an axis orientation B-scan before cornea compensation, Fig. 1(d) shows the same scan after cornea compensation. The dominating yellowish color of anterior retinal layers (Fig. 1 (c)), that was “inherited” from the cornea, is replaced by a random axis orientation, indicating that these layers are polarization maintaining. The underlying fibrotic lesion shows predominant axis orientations, varying with transversal position.

#### 2.3.5b Full compensation

To achieve full compensation for cornea and HFL/RNFL, we used the IS/OS (or lesion surface) as a reference (solid red line in Fig. 1(a)). A fully compensated example B-scan is shown in Fig. 1(e).

#### 2.3.6 Calculation of depth-averaged axis orientation map

After full compensation, the axis orientation of tissues below the reference surface should be random, with exception of fibrotic lesions that are to be segmented. Since the exact depth position of fibrotic lesions is not known, we integrate the axis orientation data along each A-scan in depth and calculate the average in complex-space, obtaining a map of averaged axis orientations. The depth integration also helps to reduce the noise. The integration is carried out between the reference surface (IS/OS or lesion surface) and a boundary that lies anterior to the sclera (since the sclera is also birefringent, it would mimic a fibrosis and therefore has to be excluded). This deeper integration boundary was empirically approximated by a 2D surface that is located 100 pixels beneath the reference surface in the macular area and 50 pixels beneath the reference surface at the image area edges (because the distance between posterior retina and sclera is larger in the macular area, more data points can be integrated here, still avoiding the sclera. It should be noted here that a less empirical way to estimate the lower integration boundary to exclude the sclera would be to segment the RPE using DOPU. However, this approach requires a complete RPE, which is not the case for many nAMD eyes). The transition of the integration boundaries between the central and peripheral parts of the imaged area was approximated by a 2D Gaussian surface. The dotted red line in Fig. 1(a) displays the second integration boundary (the integration was carried out between the solid and dotted red line). To further reduce noise, data from the depolarizing RPE were excluded from the integration (all data points with DOPU < 0.8), as well as data points where the corresponding intensity value was too low to provide reliable polarization data (values below two times the noise level of the intensity).

Figure 1(f)–(h) show the effects of compensation on the depth-averaged axis orientation maps. Figure 1(f) shows the uncompensated map. The dominating yellowish color surrounding the central area indicates the “inherited” corneal birefringent axis orientation. In the central area, a birefringent lesion is observable, identified by small patches of dominant color, indicating various components of the lesion with different local axis orientation. However, the true size and the boundaries of the lesion are unclear. After corneal compensation only (Fig. 1(g)), the corneal influence is eliminated, the lesion area is better defined, but there are still dominating colors surrounding the lesion, caused by HFL and RNFL, that make it difficult to isolate the lesion in the center. After full compensation (Fig. 1(h)), only the lesion in the central area shows predominant colors, varying with local axis orientations within the fibrotic tissue. The surrounding areas show random colors, varying from pixel to pixel, indicating non-birefringent tissue in these areas.

#### 2.3.7 Identification of fibrotic (non-random) areas

In a next step, fibrotic tissue is automatically identified and segmented by searching for areas of non-random axis orientation. The following two sub-processing steps perform this task, taking the fully compensated, depth-averaged axis orientation maps (Fig. 1(h)) as input.

#### 2.3.7a Calculation of axis uniformity

As a measure of randomness of the axis orientation, a variance map was calculated as follows: for each pixel of the axis orientation map, a circular variance in a neighborhood sized 5 × 5 pixels (corresponding to approximately 4700 µm*^2^* in a standard eye) was calculated and assigned to the central pixel. If the axis orientation values are interpreted as the angles of unit vectors, the circular variance is defined as one minus the length of the mean resultant vector computed by averaging the axis orientation vectors. The resulting variance is a unitless value between 0 (all vectors pointing in the same direction) and 1 (all vectors pointing in opposite directions). (Note that to calculate the variance, the axis orientation values were doubled in order to cover the full circle.) The variance map was filtered for low variance regions (with an upper variance threshold of th_1_ = 0.21) larger than 100 pixels (corresponding to approximately 0.02 mm^2^ in a standard eye).

#### 2.3.7b Final segmentation by region growing

The low variance regions served as seed in a region growing algorithm [56], during which the neighboring pixels were iteratively added to the segmented fibrotic region if their corresponding variance was below a second threshold th_2_ = 0.36. Both thresholds (th_1_ and th_2_) were set empirically to fit the segmented area to manual segmentation results obtained by an expert reader who used CFP and PS-OCT data as an input.

The selected regions were projected onto a pseudo scanning laser ophthalmoscopy **(**SLO) en-face map (OCT intensity projection image), marked in Fig. 1(i) in blue.

#### 2.3.8 Area calculation

In a preparatory step, the grader selects a region of interest by drawing a line around the segmented lesion that excludes artifacts, e. g. caused by the scleral canal in the vicinity of the ONH (implemented in a custom Matlab script). To quantify the segmented area, the selected pixels (Fig. 1(i)) were counted and converted to square-millimeters. With the 1024 × 250-pixel maps covering an area of 8 × 6 mm^2^, one pixel corresponds to 187.5 µm^2^ (assuming an eye of standard length).

The algorithm has been implemented in a custom Matlab script (R2019a) which was run on a computer with a 3.07 GHz Intel Core i7 CPU, maximum RAM of 12 GB and Windows 10 as the operating system.

## 3. Results

### 3.1 Fibrosis maps

**Fig. 2.**
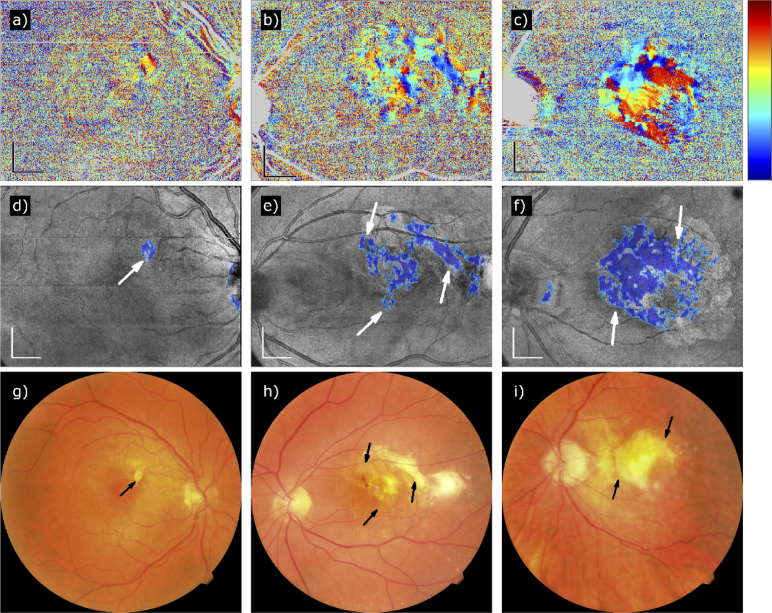
Three representative examples of PS-OCT data sets recorded in patients with fibrosis secondary to nAMD (from left to right: Patients 38, 40, and 55). (a to c) Fully compensated axis orientation en-face maps. (d to f) Fibrosis segmentation maps of the same patients. The detected fibrosis has been marked in blue on a pseudo-SLO map. (g to i) CFP of the same patients. The arrows point to regions which have been detected by our method. Color scheme of axis orientation maps: –90° to +90°. Scale bars: 1 mm.

The fully compensated axis orientation data of all 57 study eyes were used to generate en-face axis orientation maps and maps of segmented fibrosis. Figure 2 shows three representative cases: a small fibrotic area in the nasal superior region of the macula of a right eye (Fig. 2(a),(d)), a larger fibrosis at the center of the macula of a left eye, spreading temporally to the edge of the image (Fig. 2(b),(e)), and a large central fibrosis of a left eye (Fig. 2(c),(f)).

**Fig. 3.**
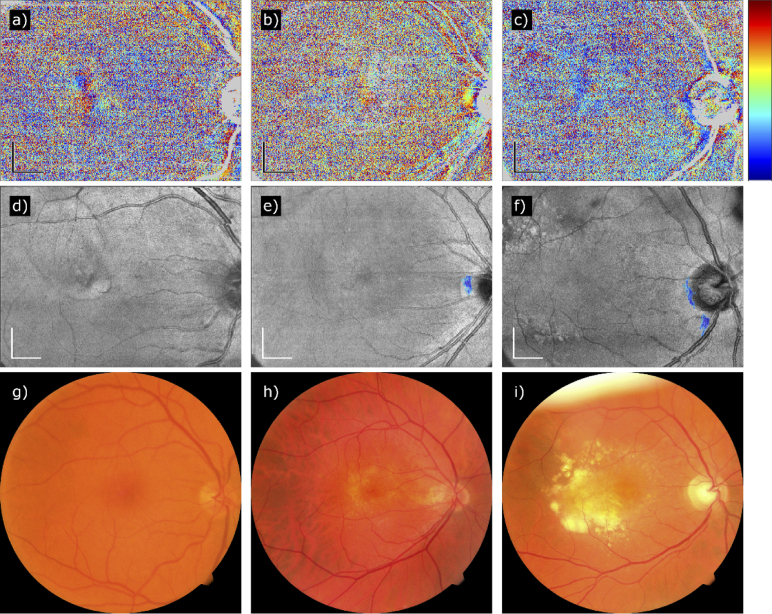
Three representative cases of PS-OCT data sets recorded in nAMD patients who did not develop any fibrotic tissue (from left to right: Patients 41, 43, and 44). (a to c) Fully compensated axis orientation en-face maps. (d to f) Fibrosis segmentation maps of the same patients. (g to i) CFP of the same patients. The blue areas in (e) and (f) are artifacts caused by scleral tissue next to the ONH. The strong discoloration in (i) is probably due to fibrin and drusen. Color scheme of axis orientation maps: –90° to +90°. Scale bars: 1 mm.

Three representative cases of nAMD patients without fibrosis are shown in Fig. 3. No fibrosis has been segmented at the central part of the map, but a few artifacts appear in Fig. 3(e) and (f) close to the ONH due to the birefringent scleral ring. The CFP in Fig. 3(g)–(i) show no discoloration due to fibrosis (in particular, the discoloration in Fig. 3(i) appears soft and not well circumscribed and was more likely caused by fibrin, and the smaller white dots represent most likely crystalline drusen).

### 3.2 Quantification and repeatability

For all 57 eyes, the areas detected by our algorithm are shown in Table 1. If more than one measurement was available, the mean of all measurements taken on the same day was calculated. The diagnoses based on both CFP and our PS-OCT algorithm are also displayed, and the discrepancies are marked in gray.

**Table 1.**
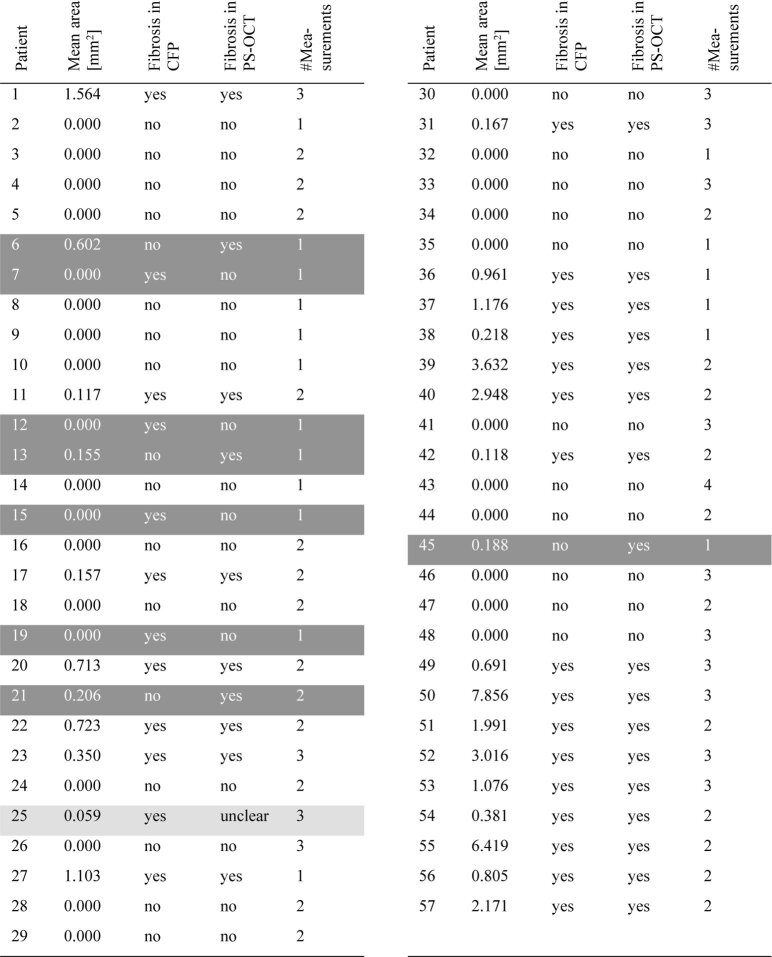
Mean lesion areas detected by our PS-OCT algorithm of all eyes and their diagnosis (yes = presence of fibrosis, no = absence of fibrosis) based on CFP and PS-OCT. The cases marked in dark gray correspond to a disagreement between the two diagnostic methods, the case marked in light gray to an inconclusive case.

**Fig. 4.**
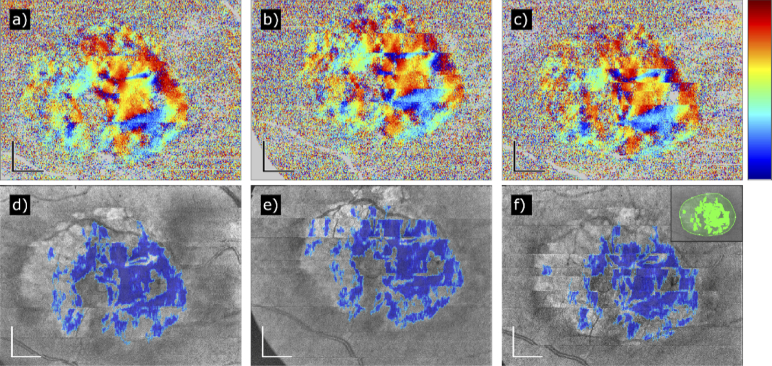
Repeatability of fibrosis segmentation. Three measurements of the same eye (Patient 50) taken on the same day. (a to c) Fully compensated axis orientation en-face maps. (d to f) Fibrosis segmentation maps. The detected fibrosis has been marked in blue on a pseudo-SLO map. The miniature in (f) shows an example of the manual selection of the region of interest. Color scheme of axis orientation maps: –90° to +90°. Scale bars: 1 mm. Detected areas: (d) 7.94 mm^2^; (e) 8.57 mm^2^; (f) 7.05 mm^2^ (mean: 7.856 mm^2^, standard deviation: 0.621 mm^2^ [8%]).

In total, 15 eyes were measured three times on the same day to demonstrate the repeatability of the results. Eight of these eyes had been diagnosed with fibrosis judged on CFP. Figure 4 shows an example of three measurements of the same patient. The mean of the detected areas is 7.856 mm^2^ with a standard deviation of 0.621 mm^2^ (8%).

**Fig. 5.**
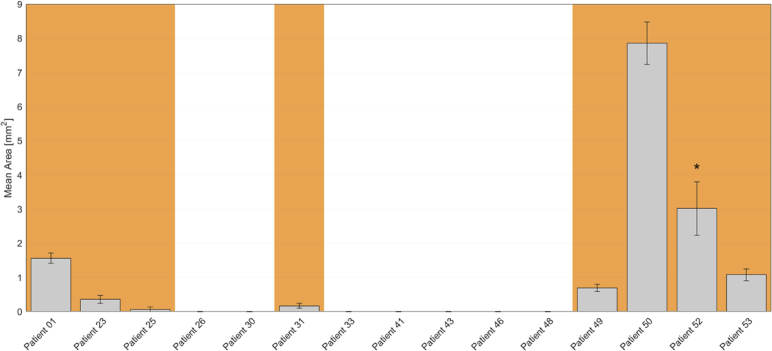
Mean areas in mm^2^ of detected fibrosis in 15 eyes with nAMD using the PS-OCT method. The area size has been calculated within the manually selected regions of interest on the automatically derived fibrosis maps (see Fig. 4(f) for an example). The orange background behind the bars indicates the presence of fibrosis in the corresponding eye as assessed by an expert based on CFP. The error bars indicate the standard deviation. Remark: *Patient 52: One measurement suffered from significant motion artifacts, hence the large standard deviation.

Figure 5 shows the mean fibrotic area of the 15 eyes included in the repeatability study with their standard deviation (error bars). The orange background indicates a positive fibrosis diagnosis as judged by the expert based on CFP. In 14 of these 15 cases, the CFP diagnostic method and our algorithm agreed about the presence or absence of fibrosis. In one case, PS-OCT was inconclusive, showing a tiny fibrosis in one measurement and no fibrosis in the two other scans. The average standard deviation of the five cases with a detected area larger than 0.5 mm^2^ is 15%.

With respect to all 57 patients, the two diagnostic methods (PS-OCT and CFP) disagreed in 8 cases ( = 14%): In four cases, the patients were diagnosed as fibrotic based on CFP but the automatic algorithm detected no fibrosis. In the other four cases, the CFP didn’t show fibrosis but the PS-OCT algorithm detected some fibrotic areas (Table 1).

**Fig. 6.**
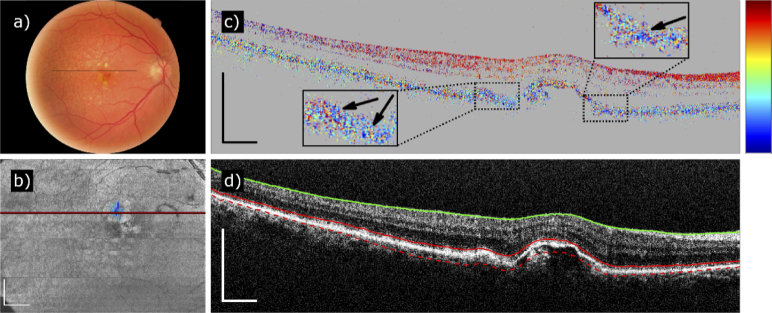
PS-OCT based fibrosis segmentation of a dataset which has been diagnosed as non-fibrotic based on CFP (Patient 45). (a) Color fundus photography. (b) Fibrosis segmentation map with the segmented areas in blue. (c) B-scan of the fully compensated axis orientation going through the segmented area. The arrows point to small regions of predominant axis value of around –70°. The trace of the B-scan is marked in (a) and (b). (d) Intensity B-scan at the same position with layer segmentation lines. Color scheme of axis orientation: –90° to +90°. Scale bar of the fibrosis map: 1 mm. Scale bars of the B-scans: 500 µm.

An example of a disagreement between the two diagnostics is displayed in Fig. 6. The CFP is lacking typical yellowish-whitish discolorations indicating the presence of fibrosis (Fig. 6(a)), yet our algorithm detected a small fibrotic region superior to the macula (Fig. 6(b)). A closer inspection of the fully compensated axis orientation B-scan reveals a borderline case around a small RPE atrophy: nasal and temporal to the atrophy, a region of a predominant axis value of around –70° can be observed (Fig. 6(c)). The locally defined axis orientation could be mere coincidence or indicate the presence of a very small or only just developing fibrotic lesion. The presence of fibrosis is therefore difficult to conclude.

**Fig. 7.**
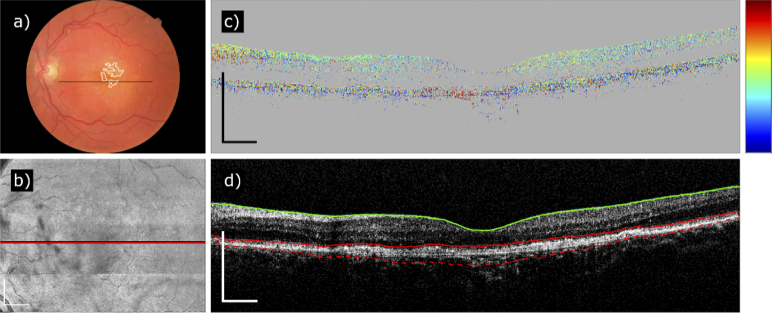
Dataset of the left eye of an nAMD patient who has been diagnosed with fibrosis based on CFP but not confirmed by the PS-OCT algorithm (Patient 25). (a) Color fundus photography. The white outlines mark yellow discolorations which were interpreted as possible fibrosis by the expert reader. (b) Fibrosis segmentation map without any segmented areas. (c) B-scan of the fully compensated axis orientation going through the area suspected to be fibrotic on CFP. The trace of the B-scan is marked in (a) and (b). (d) Intensity B-scan at the same position with layer segmentation lines. Color scheme of axis orientation: –90° to +90°. Scale bar of the fibrosis map: 1 mm. Scale bars of the B-scans: 500 µm.

The next case in Fig. 7 shows an example of the inconclusive PS-OCT case, where no fibrotic tissue was detected by PS-OCT (Fig. 7(b)), while some characteristic discoloration around the macula can be seen in the CFP. Inspecting the B-scans at the questionable position, a slight prevalence of a dominating color (orange-red) can be seen (Fig. 7(c)), however, the overlying noise is too strong for the algorithm to segment the area, so the case remains borderline.

Manual B-scan inspection of the data of the eight disagreeing cases suggest one false negative and possibly one false positive case by the PS-OCT segmentation algorithm. Many cases were however difficult to conclude even manually, depending on the quality of the scan.

## 4. Discussion

Currently fibrosis is diagnosed by expert readers by evaluating color fundus photography, fluorescein angiography, and standard OCT, which is subjective and often inconclusive, because fibrotic patterns can be irregular and visual clues given by the aforementioned imaging techniques may be ambiguous. Taking advantage of the birefringent properties of fibrotic tissues, PS-OCT can provide additional contrast and allow for more precise and reliable evaluations [34,35,37]. However, previous methods had problems with smaller fibrotic lesions that generate low cumulative retardation, being disturbed by influences of overlying birefringent tissue like the RNFL or HFL [34,35], or required time-consuming visual inspection of B-scans of different contrast modalities [37]. In this work, we developed a new automatic detection algorithm which greatly reduces the work load of expert readers and may provide a faster diagnosis.

Our algorithm works on PS-OCT volume data sets and calculates depth-averaged axis orientation maps of the posterior retina. It is based on the fact that the birefringent fibrotic tissue exhibits areas of well-defined birefringent axis orientation, typically consisting of patches whose optic axis locally points in the same direction. The surrounding non-birefringent tissue exhibits, after compensation of anterior birefringent structures, a random axis orientation, varying from pixel to pixel. By calculating maps of local axis variance, the uniformity of the optic axis is assessed and used to segment fibrotic areas. Our algorithm requires minimal user interaction, using the same axis variance threshold values for all investigated data sets. The only user interaction required is the definition of a region-of-interest where the algorithm searches for fibrosis. This takes only a few seconds and is required to exclude the problematic areas in the vicinity of the ONH, where scleral signals can disturb the algorithm (this might be improved by future versions of the algorithm that automatically detect and exclude the ONH region).

It should be mentioned here that the successful outcome of the algorithm is strongly dependent on the correct segmentation of the tissue boundary below HFL. This layer segmentation line defines the surface that serves as reference to compensate for the birefringence of overlying tissues as well as the volume for which the en-face maps are computed. Other PS-OCT based methods that measure depth-resolved, local retardation and axis orientation could also be used. The majority of such algorithms require the use of at least two different polarization states in the sample and/or the reference arm (see, e.g. [27,37,43,48,57,58]), which would make the instrument more complicated. One of these methods has already been demonstrated for identifying fibrotic tissue in the retina [37], however, an automated way to quantify lesion size based on this technique has not yet been reported.

Also single-input-state methods have been reported for local, depth resolved birefringence analysis [59,60]. In one of these studies [59], local birefringence was obtained by an iterative algorithm that calculates local axis orientation and local retardation pixel by pixel along the depth coordinate. It has been shown that such an iterative method is required for a mathematically exact extraction of local axis orientation because the use of a single step compensation, while being sufficient for local retardation analysis, does not account for an additional, unknown offset of the axis orientation [61] that depends on retardation and axis of the anterior layers. However, for realistic scenarios encountered in our study, the differences between exact axis orientation and the single-step compensation approximation are negligible: Assuming a typical corneal retardation of δ_C_ ∼ 20° and a maximum retardation for HFL of δ_HFL_ = 14° [30], the maximum deviation between true and measured axis orientation is ∼1.3°. (The additional constant axis offset introduced by the PM fibers is ignored here since it just rotates the entire axis pattern without influencing variances). Moreover, the deviation between exact and approximate value varies only gradually with position along a circle around the foveal center. We simulated this effect and found that, within the 5 × 5 pixels evaluation kernel used to calculate the axis variance, the additional variance introduced by the approximation is < 10^−5^. This is negligible compared to the variance threshold of 0.21, used for the fibrosis segmentation. (For comparison, we also carried out a two-step iterative compensation [step 1: cornea compensation with ILM as reference surface; step 2: HFL compensation with IS/OS or lesion surface as reference]; the results were very similar to those of the single step compensation, so we omit them here; very small deviations in some borderline cases were likely caused by the additional ILM segmentation step and the associated segmentation inaccuracies and noise.). However, an iterative compensation strategy like that used in Ref. [59] might be useful to further analyze whether there are axis orientation changes along the depth direction within thicker fibrotic lesions (presently, a constant axis orientation with depth is assumed for such lesions, supported by our observation of “columns” of constant axis in thicker lesions [34]).

We tested our method in 57 eyes of 57 patients with nAMD. 28 of these eyes had been diagnosed with fibrosis on corresponding CFPs by an expert reader, 29 eyes had been classified as non-fibrotic. Of the 28 fibrotic eyes on CFP, 23 were confirmed by our algorithm, one case was inconclusive on PS-OCT, the remaining 4 eyes did not show signs of fibrosis on PS-OCT. 25 of the 29 non-fibrotic eyes on CFP were confirmed by PS-OCT, in the remaining 4 eyes, small fibrotic areas were identified by PS-OCT. Table 1 shows a comparison of fibrosis detection by CFP and PS-OCT across all cases, and lists the lesion size measured by PS-OCT. The eight disagreeing cases are marked in dark gray, the inconclusive case is marked in light gray.

It can be observed that in the four disagreeing cases which were diagnosed as non-fibrotic based on CFP, our PS-OCT algorithm only detected small lesion areas. All were smaller than 0.7 mm^2^, three of them 0.2 mm^2^ or smaller. This should be set into relation with the lower detection limit of the algorithm, which requires at least 100 contiguous pixels (0.019 mm^2^ for a standard eye) within the fibrotic lesion. For very small lesions, the algorithm might therefore be somewhat unreliable.

To better understand the properties of a segmentation algorithm, we have to analyze its precision and accuracy. To evaluate the precision (repeatability) of our method, we recorded three successive data sets in a subset of 15 eyes (on the same day, each). Eight of these eyes had been diagnosed with fibrosis on CFP, in seven cases, no fibrosis was observed on CFP. PS-OCT confirmed these diagnoses in 14 cases, one case was inconclusive. In the eight cases where fibrosis was detected with PS-OCT, mean ± SD was calculated for each case. We observed a wide range of lesion sizes, ranging from 0.06 to 7.86 mm^2^ (cf. Table 1). The precision (repeatability) of lesion sizes > 0.7 mm^2^ ranges from 8 to 26%, being 15% on average. In one case (patient no. 52), one measurement suffered from larger motion artifacts, leading to an SD of 26% for this case. If we exclude this case, the mean precision of measurements was 12% for cases with nearly motion artifact free data (this value is likely to be improved by recording data sets with our recently reported re-take function [39], that automatically re-records data in areas that had been corrupted by larger motion artifacts or eye-blinks; only part of the data reported here have been taken with this function implemented).

Due to lack of a true gold standard, it is difficult to assess the presence of fibrosis with absolute certainty, and therefore it is also challenging to estimate the correctness (accuracy) of the algorithm. However, we can discuss the properties of our algorithm, and the conditions that lead to its decision to designate a pixel as fibrotic or not, in some more detail.

**Fig. 8.**
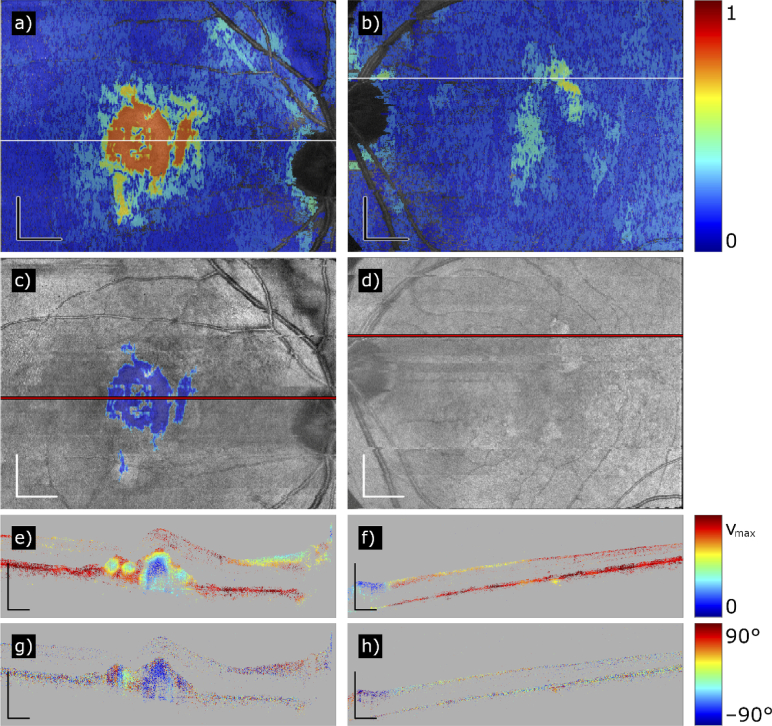
Maps that illustrate the interpretation of the fibrosis segmentation maps for two different cases. (a) Likelihood map of a case with severe fibrosis (Patient 39). The colors correspond to the likelihood of presence of fibrotic tissues. (b) Likelihood map of an ambiguous case (Patient 2). (c) Fibrosis segmentation map of case (a). (d) Fibrosis segmentation map of case (b). (e) Variance B-scan (trace marked in (a) and (c)) of case (a). (f) Variance B-scan (trace marked in (b) and (d)) of case (b). (g) Fully compensated axis orientation B-scan of case (a) (trace marked in (a) and (c)). (h) Fully compensated axis orientation B-scan of case (b) (trace marked in (b) and (d)). Color scheme of the likelihood maps: 0 to 1. Color scheme of the variance maps: 0 to v_max_. Color scheme of the axis orientation B-scans: –90° to +90°. Scale bars of the en-face maps: 1 mm. Scale bars of the B-scans: 500 µm.

In particular, whether a region of the variance map of the average axis orientation en-face map is interpreted by the algorithm as sufficiently well-defined axis orientation, that is, corresponding to a lesion, is dependent on two thresholds: a global one to define the seeds for a region growing algorithm (th_1_, set to 0.21 in this study) and a local one which determines whether neighboring pixels of the growing region are added to the lesion area (th_2_, set to 0.36 in this study). It is difficult to set the thresholds in a way to both detect weak fibrosis and avoid false positives. One could approach this problem by running the algorithm with a range of thresholds and create, based on the strictest threshold that results in detected fibrosis, a likelihood map for the occurrence of fibrosis. Two examples of such maps can be seen in Fig. 8(a) and (b). For these maps, the algorithm was run 18 times with th_1_ varying between 0 and 0.85 and th_2_ always set to th_1_ + 0.15. The color values on the map indicate the likelihood L_h_ of a pixel corresponding to fibrosis (blue: low likelihood; red: high likelihood; L_h_ = 1 – th_2_, with th_2_ indicating the lowest threshold that lead the algorithm to segment that pixel as fibrotic). Regions that are detected with a lower threshold are more likely to be fibrotic than regions that are only detected with very high thresholds: Fig. 8(a) shows a region centered at the macula which is already detected with a th_1_ of 0.05 and a th_2_ of 0.2 (red), which is very likely to be fibrotic, surrounded by areas of th_1_ = 0.25 / th_2_ = 0.4 (yellow-green), which still might be fibrotic but the outcome of the algorithm is less reliable (compare to the regions that actually got segmented by our algorithm, shown in Fig. 8(c)). Figure 8(b) shows a borderline case with a small region requiring rather high thresholds of th_1_ = 0.25 / th_2_ = 0.4 (yellow-green) to be segmented. Indeed, a closer inspection of the B-scans in that region revealed the possible but not definite presence of a weak fibrosis (cf. the fibrosis segmentation map in Fig. 8(d) that does just not segment the questionable area (because the segmentation map requires th_1_ ≤ 0.21, but the required threshold for this case would be th_1_ = 0.25) and the fully compensated axis orientation B-scan in Fig. 8(h)).

In addition, the axis uniformity not only manifests in en-face maps but also in depth: axis orientation B-scans of severely fibrotic eyes show a column-like structure due to projections on structures located posterior to the fibrous tissue [34]. This effect is clearly visible in severe fibrosis (Fig. 8(g)) and less pronounced in weak fibroses, and can be quantified by calculating variance B-scans of the fully compensated axis orientation B-scans: for each pixel, the variance was calculated in a neighborhood of 41 × 41 pixels. Figure 8(e) and (f) show B-scans of axis variance of a severely fibrotic and a borderline case, respectively. Figure 8(e) has a lower variance in the region where fibrosis was detected with the algorithm, implying a more uniform in-depth axis orientation (cf. Figure 8(g)). In contrast, the variance of the case in Fig. 8(f) remains elevated almost everywhere (with exception of a tiny region in the area of the presumptive lesion), which is reflected in the random axis orientation in Fig. 8(h).

The maps displayed in Fig. 8 will not provide clear diagnoses but are additional tools for an expert to conclude on the presence or absence of fibrosis. It remains to be seen whether ophthalmologists and retina specialists prefer these likelihood maps over a simpler yes/no segmentation or not.

## 5. Conclusion

We developed a new method to automatically detect and quantify fibrotic lesions in the retina of patients with neovascular age-related macular degeneration. The method is based on polarization-sensitive OCT and exploits the birefringence of the fibrotic tissue to reliably detect and quantify lesions larger than 0.7 mm^2^. The algorithm developed requires only minimal operator intervention and should therefore be useful for diagnostics and follow-up studies in larger patient cohorts.

## Data Availability

Data underlying the results presented in this paper are not publicly available at this time but may be obtained from the authors upon reasonable request.
